# Supplementary figures and images for: Difluoromethylornithine Induces Apoptosis through Regulation of AP-1 Signaling via JNK Phosphorylation in Epithelial Ovarian Cancer
Source: Int J Mol Sci. 2021 Sep 23;22(19):10255. doi: 10.3390/ijms221910255 (PMC8508876; doi:10.3390/ijms221910255)

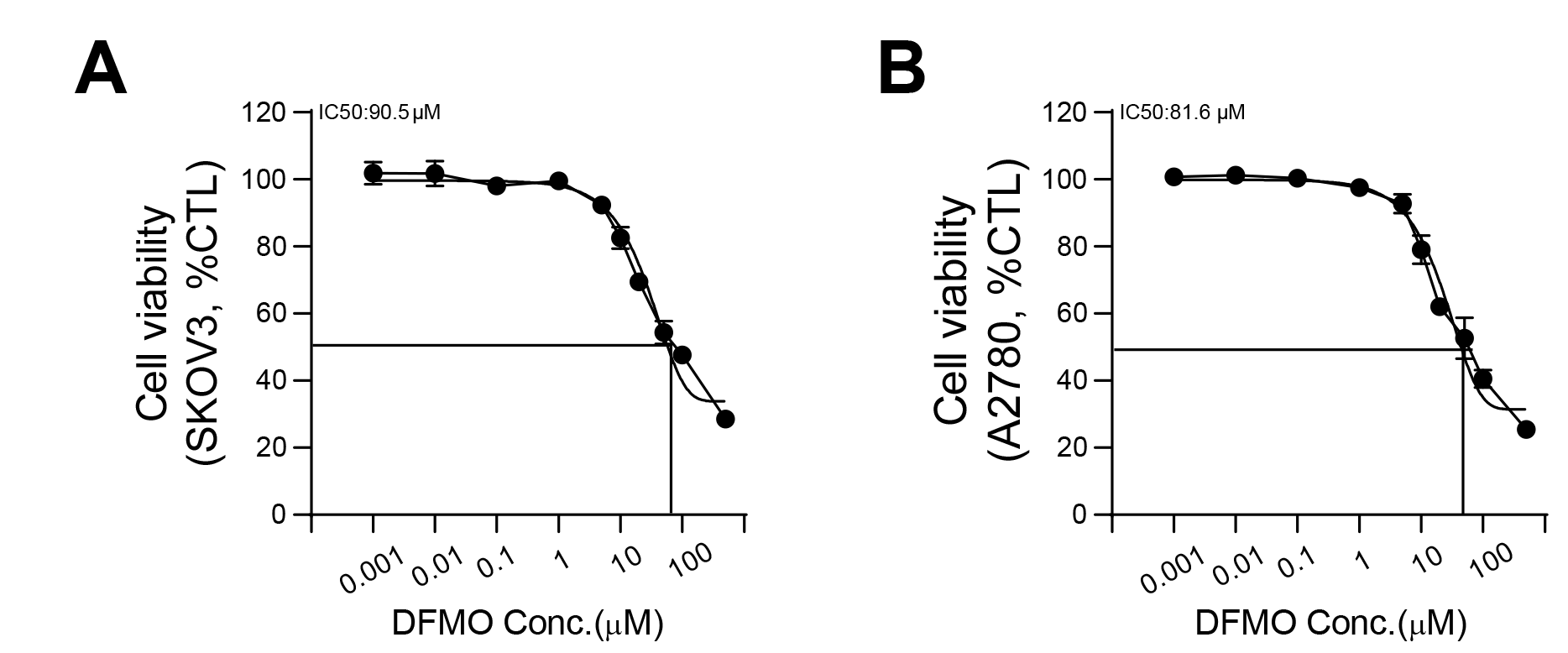

Supplement: Supplementary file 1 [file ijms-22-10255-s001.zip › supplemental Figure S1.tif]

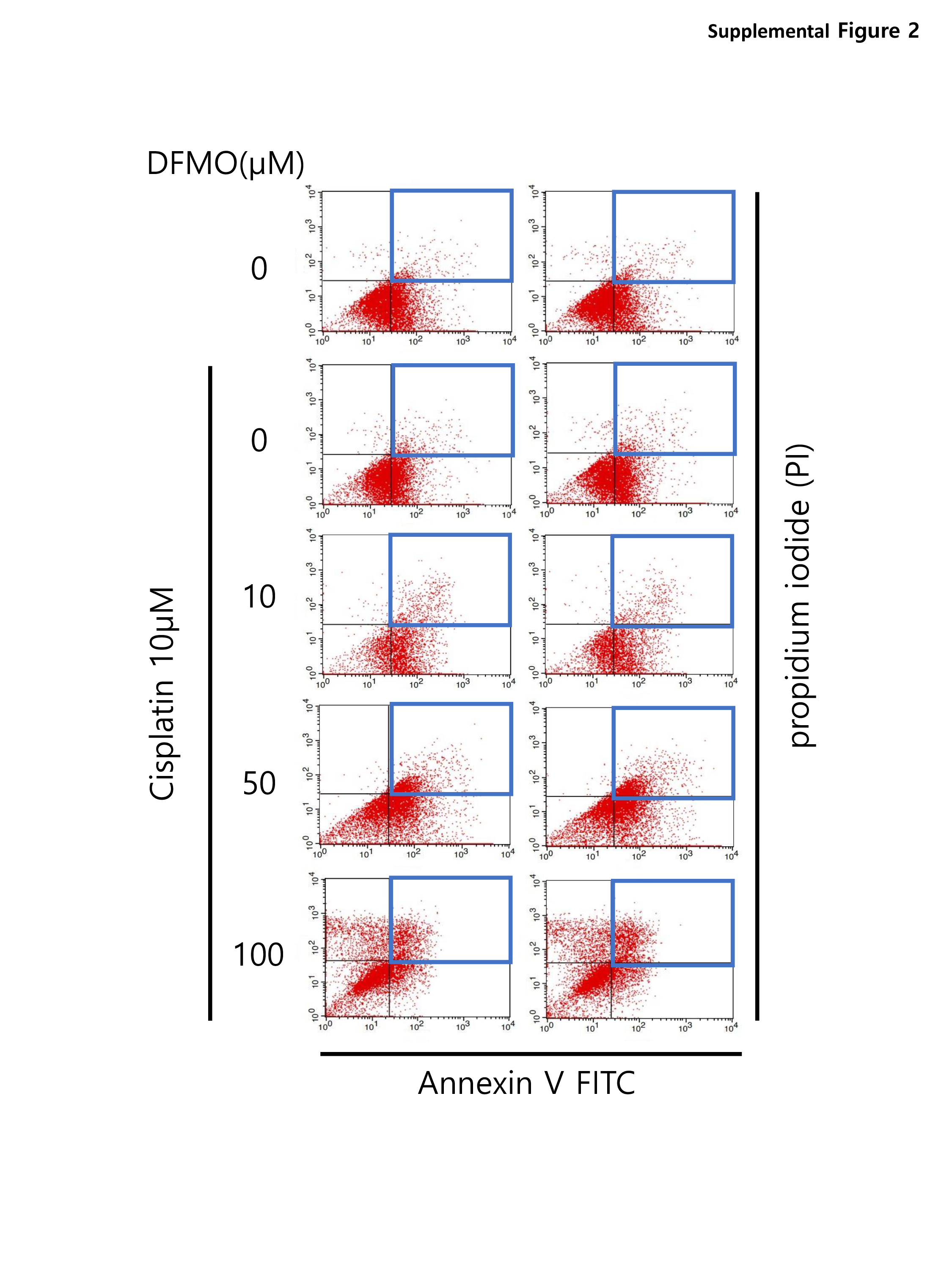

Supplement: Supplementary file 1 [file ijms-22-10255-s001.zip › supplemental Figure S2.tif]
